# Improved recovery time and sensitivity to H_2_ and NH_3_ at room temperature with SnO_x_ vertical nanopillars on ITO

**DOI:** 10.1038/s41598-018-28298-w

**Published:** 2018-07-03

**Authors:** L. D’Arsié, V. Alijani, S. T. Suran Brunelli, F. Rigoni, G. Di Santo, M. Caputo, M. Panighel, S. Freddi, L. Sangaletti, A. Goldoni

**Affiliations:** 1 Elettra – Sincrotrone Trieste S.C.p.A., s.s. 14 km 163.5 in Area Science Park, 34149 Trieste, Italy; 2 Department of Engineering, University of Cambridge, Cambridge, CB3 0FA United Kingdom; 3 Interdisciplinary Laboratory for Advanced Materials Physics and Dipartimento di Matematica e Fisica, Università Cattolica del Sacro Cuore, Brescia, Italy; 4 Università degli Studi di Trieste, Piazzale Europa 1, 34127 Trieste, Italy

## Abstract

Nanostructured SnO_2_ is a promising material for the scalable production of portable gas sensors. To fully exploit their potential, these gas sensors need a faster recovery rate and higher sensitivity at room temperature than the current state of the art. Here we demonstrate a chemiresistive gas sensor based on vertical SnO_x_ nanopillars, capable of sensing < 5 ppm of H_2_ at room temperature and 10 ppt at 230 °C. We test the sample both in vacuum and in air and observe an exceptional improvement in the performance compared to commercially available gas sensors. In particular, the recovery time for sensing NH_3_ at room temperature is more than one order of magnitude faster than a commercial SnO_2_ sensor. The sensor shows an unique combination of high sensitivity and fast recovery time, matching the requirements on materials expected to foster widespread use of portable and affordable gas sensors.

## Introduction

Gas sensors are indispensable in a modern society. Sensing H_2_, for instance, is increasingly important, as this molecule is envisaged as an alternative to fossil energy sources^1^. For a safe transport and storage of H_2_ to be viable, safety needs to be guaranteed, and therefore appropriate sensing for leakages^1^. In environmental monitoring, it is key to measure trace amounts also of other gases, such as CO, NO_2_ and NH_3_^2,3^. Indoors it is desirable to use simple and inexpensive sensors for detecting NO_2_ and CO_2_^3,4^, to monitor air quality. Moreover, gas sensors can find applications in exams of medical conditions, for example through breath tests^5^, as well as helping food quality control or contamination detection^6^. These are some of the many important applications of gas sensing, which explain the intense research in the field.

Today nanoscience allows the development of metal oxide sensing materials, promising dramatic changes in design and capabilities^7^. These improvements include the possibility to drastically reduce the size, weight, and power consumption, while increasing sensitivity and selectivity^8,9^. The widespread use of chemiresistive sensors is favoured by the ease of manufacturing requirements and low cost^10–13^, in spite of a poor selectivity^14^. Chemiresistive sensors are therefore commercially very attractive, opposed to electrochemical sensors, which are unpopular for their short lifetime, and optical sensors, which are generally more expensive and bulky. A chemiresistive gas sensor consists of a sensitive material capable of changing its electrical properties when molecules of a certain gas are adsorbed on its surface. Tin dioxide (SnO_2_), a wide band-gap semiconductor with remarkable chemical stability and electrical properties, is the most studied among semiconducting metal oxides for gas sensing applications^15,16^. Even if the selectivity is generally low, SnO_2_ has demonstrated impressive sensitivity for a large range of types of gas. There is therefore strong interests in developing strategies aimed at enhancing the SnO_2_-based device performance and selectivity. These include modifying the material morphology at the micro- and nano-scale, the bulk chemical composistion (i.e. doping), and by engineering the surface^17–21^. For instance, nanoparticles, nanowires, nanotubes and other nanoscale morphologies have enhanced gas-sensing properties^22,23^, as a high surface area-to-volume ratio is key for enhancing the response due to the adsorption of molecules. Moreover, SnO_x_ chemiresistive sensors, when compared to competing sensing materials and techniques, are generally less expensive, lighter, more robust, and more sensitive to a wider range of gasses with quicker sensing and recovery times.

In order to fully develop SnO_x_ sensors, two main targets should be reached, namely short recovery time and sensitivity in the ppb range. Short recovery time is needed in situations of rapid modifications of gas concentrations. Whereas high sensitivity is necessary in applications that require the detection of low concentration gases (down to the ppt range), for instance in biochemical processes occurring in the human body and detected by analysing the breath of patients^5^. SnO_2_ chemiresistors typically work in the temperature range 200–400 °C with a sensitivity that depends on the analyte gas, between 30 ppb and 500 ppm^15,24^. The recovery time is in the order of tens of seconds at high-temperature and often increases to several minutes at room temperature (RT). Elevated working temperatures are deleterious for energy consumption and therefore portability, as well as for safety during explosive gas detection, making efficient gas sensing at RT a highly desirable target.

Here we demonstrate that a chemiresistor based on vertical SnO_x_ nanopillars grown on Sn-reduced indium tin oxide (ITO) thin films^25^ can detect 1 ppm of NH_3_ at RT in air, with a recovery time of seconds, i.e. about an order of magnitude faster than commercially available metal oxide chemiresistor operating in the same conditions. It also detects few ppm of H_2_ at RT in vacuum and, at high-temperature, it can recognize the equivalent of 10 ppt of H_2_ (10^−8^ mbar). Thanks to the ease of fabrication and the efficient gas sensing characteristics, the proposed nanostructured material paves the way towards economic, safe, and portable gas sensors.

## Results and Discussion

**Figure 1:**
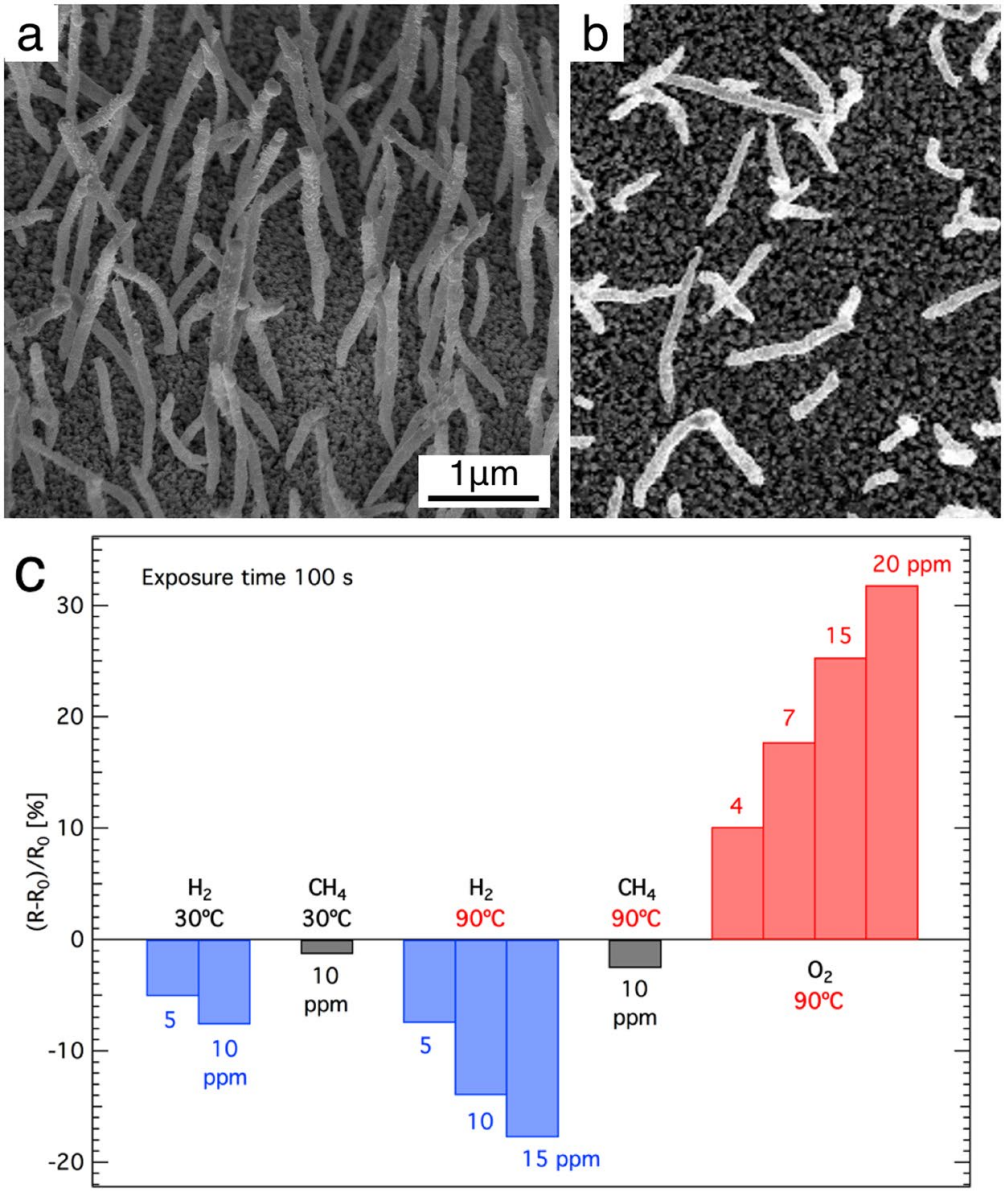
Scanning electron microscope images of vertical nanostructured SnO_x_ pillars on ~150 nm thick ITO Sn-reduced substrate. (**a**) 45° tilt angle, and (**b**) 0° tilt angle. The two figures share the same scale bar. The images are taken before starting the sensing tests and is representative of the post-sensing morphology, as no change can be noticed under the SEM. (**c**) Response of vertical nanostructured SnO_x_ pillars after exposure for 100 s to several gases at low temperatures (30 or 90 °C) in low vacuum (10^−5^ mbar).

The vertical SnO_x_ nanopillars grown on ITO are shown in Fig. 1a,b. These sensing layers display huge resistivity variations for several gasses, already after an exposure in the ppm range for 100 s at RT. Figure 1c illustrates the variation in resistance (R − R_0_)/R_0_ [%] of the sensor to H_2_, CH_4_, and O_2_ at RT and at 90 °C, where R is the resistance after 100 s of gas exposure and R_0_ is the initial resistance before the introduction of gas in the experimental chamber. The experiment is performed in low vacuum (10^−5^ mbar as base-pressure). At 90 °C and 10 ppm, we have observed resistivity variations in the range 15–20% for H_2_ and O_2_, while for CH_4_ the sensitivity is one order of magnitude lower. These percentage changes are related to the relatively high initial resistance (R_0_), which is of the order of 500 kΩ/sq. The sensor is insensitive to CO_2_ and N_2_ concentrations up to 1000 ppm. Moreover, similar treated substrates with nanometer roughness, i.e. ITO annealed at 670 ± 10 °C without any nanopillars, do no show sensitivity below 1000 ppm to all the analyte gases above indicated (see Supporting Information).

The observed behaviour can be explained by the effect of the gas molecules interacting with the n-type SnO_x_ nanopillars^26,27^. Negative charges trapped by oxidizing species generally cause an upward band bending, while the reaction with reducing gases, such as H_2_, a downwards band bending. However, it is useful to notice that competitive adsorption and replacement of the adsorbed molecules could decrease or even reverse the band bending, resulting in an opposite variation in the conductivity^26,27^.

It is important to remark that the ITO layer does not contribute appreciably to gas sensing, as discussed in the SI (Figs SI.1 and SI.2). In fact attempts to use an ITO layer without nanopillars as a sensor yielded only a variation of the order of 0.2–0.5% after the introduction of 1000 ppm of H_2_, CH_4_, O_2_, N_2_, CO_2_ per 100 s at RT.

To further investigate the sensitivity to H_2_ at high temperature, we place the sample at 230 °C in ultra-high-vacuum (UHV, 10^−10^ mbar) chamber. This allows us to expose the sensor to small amounts of gas (10^−8^). Although UHV experiments do not replicate sensor typical operational conditions, in very low-pressure experiments it is possible to analyse the sensor with a higher signal-to-noise ratio and precision. At the same time, the preparation and characterization of the sample in UHV appears to be an important step to identify and properly address the chemo-physical processes that occur at the surface of the sensor.

**Figure 2:**
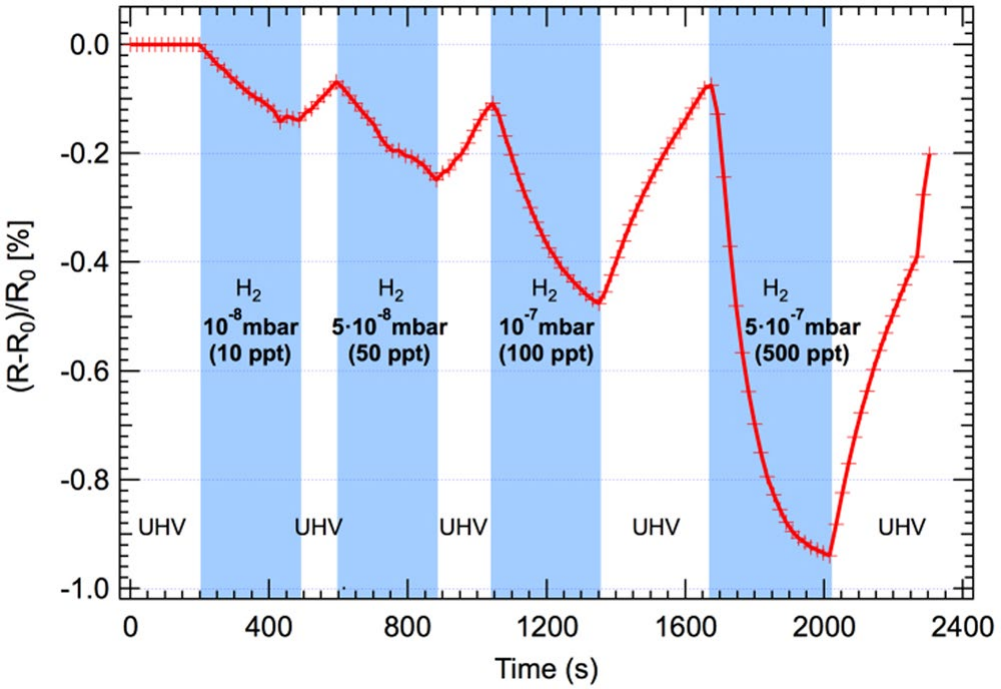
Response of SnO_x_ nanopillars sensor to low pressures of H_2_ (corresponding to ppt amount) at 230 °C in UHV.

The resistivity of the sample increases to about 3 MΩ in UHV after removing all carbon contamination by annealing at 300 °C in an oxygen atmosphere (10^−7^ mbar). Figure 2 shows the response of the SnO_x_ nanopillars from 10^−8^ mbar up to 5 × 10^−7^ mbar (ppt amounts) of H_2_, expressed as (R − R_0_)/R_0_ [%]. It can be observed that the chemiresistor is sensistive at least to 10^−8^ mbar (10 ppt) of H_2_.

It is worth noting that at these concentrations and at 230 °C the sensor does not respond to CH_4_, CO_2_, CO, NO_2_ and N_2_. The X-ray photoemission data of Sn 3d peaks collected during the 5 × 10^−7^ mbar of H_2_ exposure are reported in the Supporting Information (Fig. SI.3). The Sn 3d peaks show a shift to higher binding energy when the gas is introduced and revert back to the initial binding energy when the gas is removed and the chamber reaches the base pressure of 10^−10^ mbar, in agreement with the expected variation of the band bending. This recovery to the initial conditions observed through XPS core level analysis further confirms the reversible character of the interaction of the target molecules with the chemiresistor.

**Figure 3:**
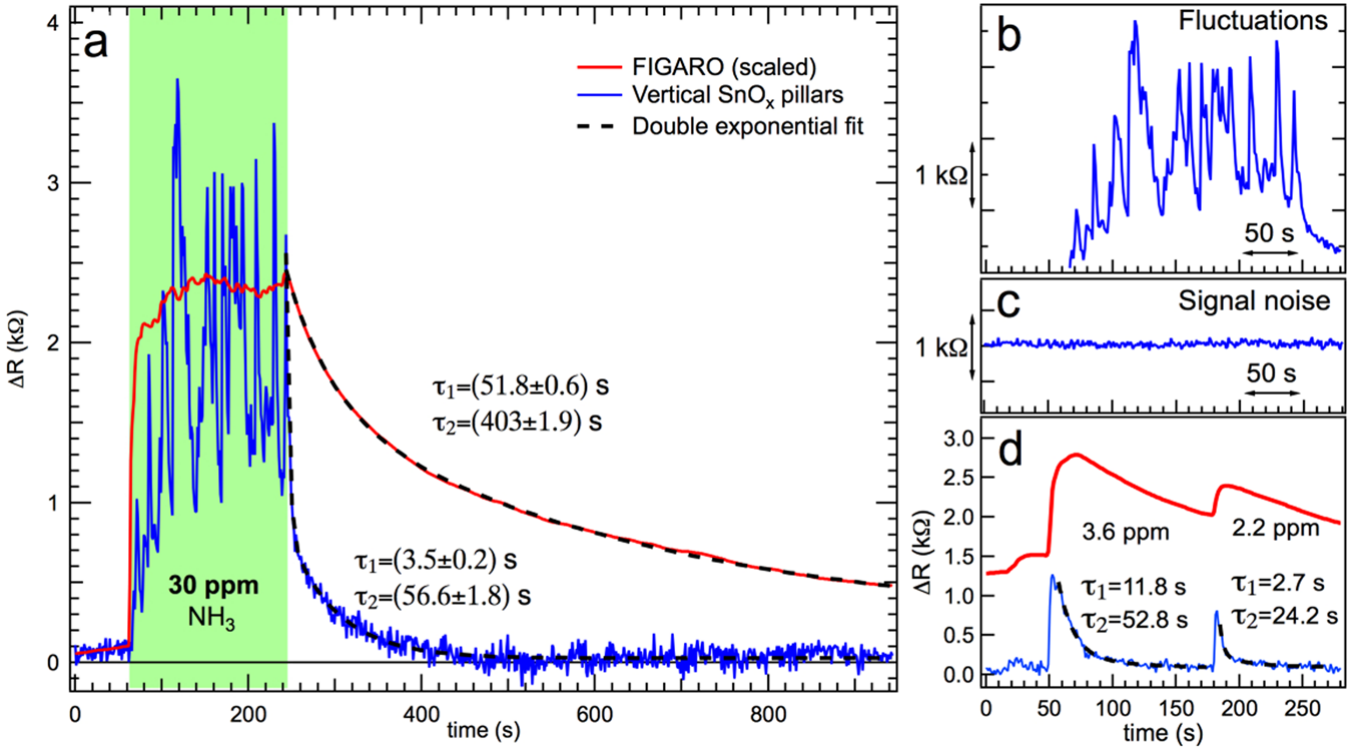
(**a**) Resistivity variation of the vertical SnO_x_ nanopillars sensor (blue line) compared with the FIGARO sensor (dashed red line) after an exposure to 30 ppm of NH_3_ in air at RT. The NH_3_ exposure period has been marked with a green background. Both recovery times are fitted with double exponential functions, illustrated by dashed black lines. (**b**) Observed fluctuations can be compared with (**c**) the signal noise. (**d**) Shows the resistivity variation of our vertical SnO_x_ nanopillars sensors (blue line) compared with the FIGARO response (dashed red line) after an exposure to 3.6 and 2.2 ppm of NH_3_ (pulse in the millisecond range).

In order to further investigate the response and the recovery time of our chemiresistive sensor, we have compared it to a commercial sensor for NH_3_ (FIGARO TGS 2602). Our sample is operated at RT, while the FIGARO requires heating at 200 °C. The same measurements performed on 4 sensors show reproducibility across different substrates, as shown in the SI (Fig. SI.4). Our sensor follows the FIGARO response, but presents more oscillations, see Fig. 3. A comparison of fluctuations and noise amplitude in of Fig. 3b,c, respectively, should suggest that this behaviour is not a read-out noise amplitude fluctuation. It can be attributed to local partial pressure variations of the order of 1 ppm or less while exposing the sensor to 30 ppm of NH_3_ from a point-like source in ambient air. This is confirmed in Fig. 3d where for exposures to a lower ammonia concentration (3.6 and 2.2 ppm), as well as for a reduced time, the fluctuations are greatly reduced. The presence of these fluctuations, however, is not related to the response time (which is very short for both SnO_x_ and FIGARO), but to the very rapid recovery time with respect to the FIGARO behaviour. As shown in Fig. 3, when the NH_3_ is pumped away, the overall recovery of our sensor is so fast that in less than 200 s the initial resistance is completely recovered, while the reference FIGARO sensor has not yet fully recovered. It is also worth noting that the recovery does not follow a simple exponential behaviour, as the process takes place with two different time scales. We therefore fit this behaviour using a decreasing double exponential function with exponential constants τ_1_ and τ_2_. The obtained recovery time gives a τ_1_ value of few seconds (3.5 s in Fig. 3a and 2.7 s in Fig. 3d) and τ_2_ of tens of seconds, both of which are about one order of magnitude lower than the same fitting procedure for the recovery time of FIGARO sensor. The presence of τ_1_ and τ_2_ in both sensors may be related to different interactions of the physisorbed NH_3_ molecules with the substrate.

The difference in recovery rates shown in Figs 2 and 3 can be attributed to the inefficient H_2_ pumping by turbo-molecular pumps and the large volume of the experimental chamber. The resistance recovery rates in Fig. 2 are a convolution of the sensor recovery and the slow pressure recovery rates. The sensing test with H_2_ in ambient air could not be performed for health and safety regulations.

In addition to fast response, also selectivity and stability are important characteristic of gas sensing. Selectivity in air has been checked by exposing the sensor to different gasses. As detailed in the SI (Fig. SI.5), sensor is not sensitive to acetone, 2-propanol, and sodium hypochlorite. Response to ethyl alcohol is quite low as compared to ammonia.

Furthermore the response to ammonia was tracked for long exposure times (250–300 seconds) and high concentrations (50–70 ppm). In spite of the prolonged interaction with large concentration of gas, full recovery was attained due to the fast recovery time (Fig. SI.6). Response to humidity was also investigated and the sensor displayed a resistance drop with humidity increase (Fig. SI.7), consistently with an n-type behaviour.

Finally, stability in ambient air for relatively long periods (24 hours) has been checked several times during our experiments. As shown in the supplementary information (Fig. SI.8), the sensors were quite stable with a base resistance that did not change across the time, unless the outer conditions (temperature or relative humidity) changed along the monitoring time.

The very fast recovery rate of our sensor is outstanding when compared with results in literature. However, other promptly responsive RT sensors have been reported^28–32^. Favier *et al*.^30^, for instance, have produced Pd nanowires sensors with a 90% signal saturation reached in less than 80 ms at RT. These fast responses are however achieved with very high H_2_ concentrations (20–100 ppm). They propose a sensing mechanism that involves the closing of nanoscopic gaps or “break junctions” caused by the dilation of wire grains. This creates a sensor that works more as an on/off switch than a continuous detection, which explains the excellent results at high concentrations, but also a high gas detection threshold (5–10 ppm). Changing the geometry of the Pd nanostructures can enhance the sensitivity to lower detection limits, although penalising recovery rates^31^.

**Figure 4:**
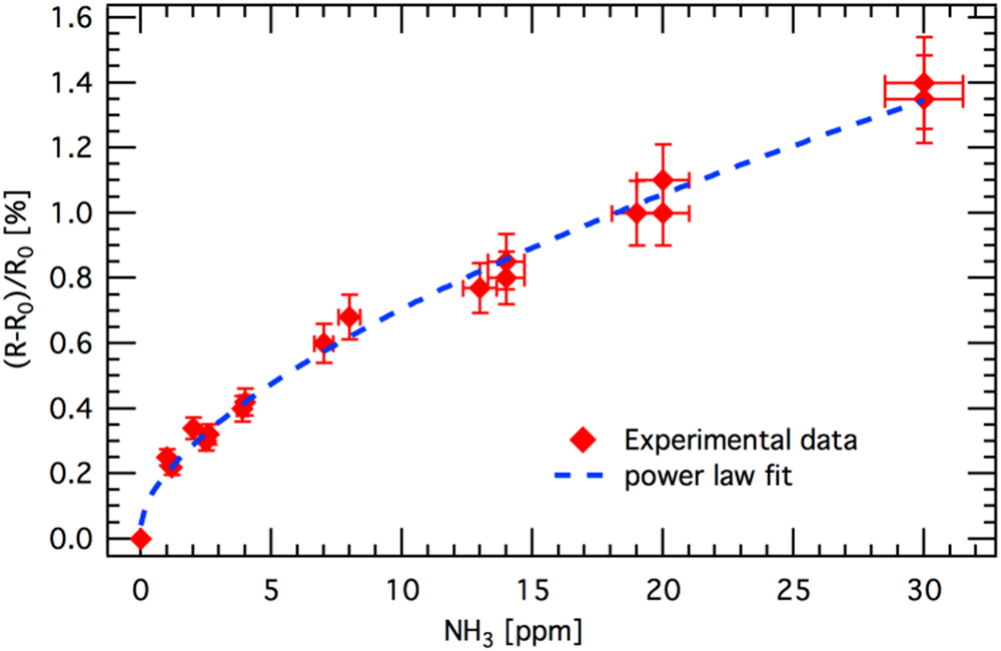
Experimental response of the sensor as a function of NH_3_ exposure at RT in air, with about 50% of relative humidity. The power law fit is meant as a guide for the eye.

Finally, our sensor can detect at least 1 ppm of NH_3_ at RT (see Fig. 4), which is comparable with recent results in literature, in which however, the sensing response/recovery rates are high. Mubeen *et al*.^33^ for instance, reported the detection of 0.5 ppm of NH_3_ at RT, using single wall carbon nanotubes decorated with tin oxide, but the response/recovery rates are in the time scale of several minutes.

**Figure 5:**
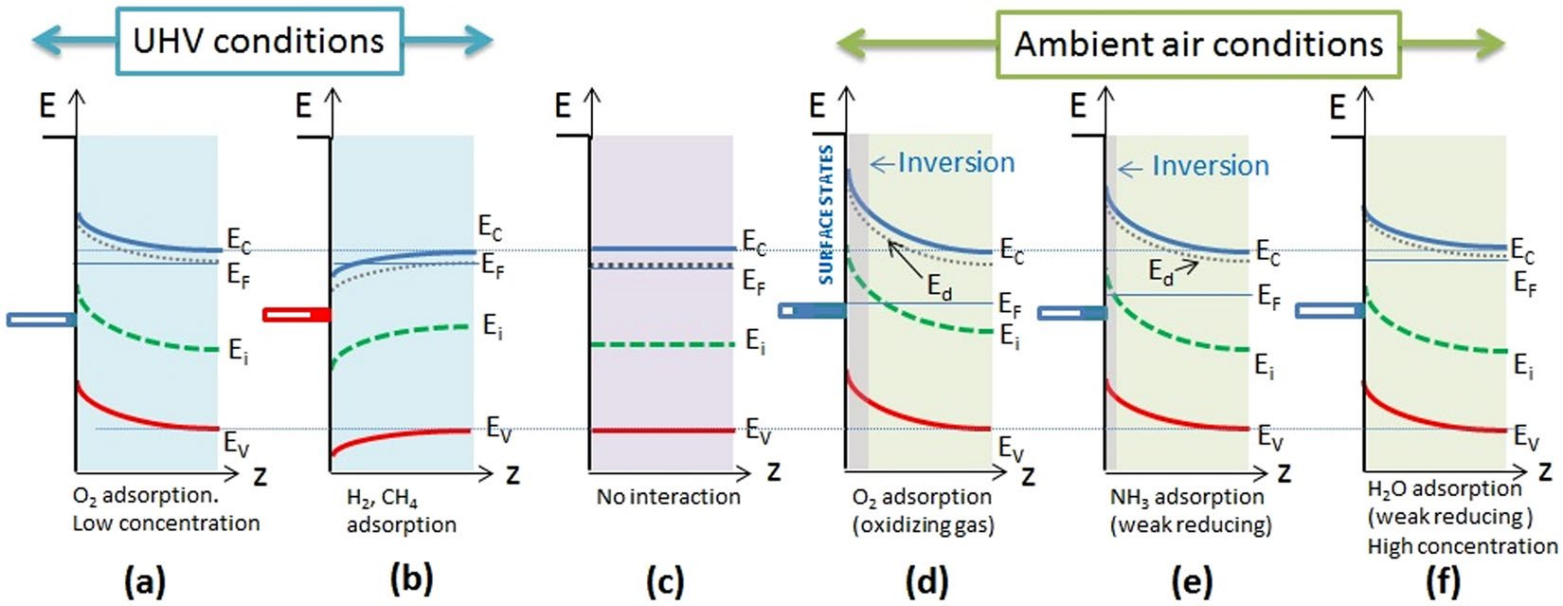
Schematic diagrams of the nanopillar electron bands under different conditions (UHV and ambient air) after exposure to different gases. (**a**) O_2_ in UHV, (**b**) H_2_ or CH_4_ in UHV, (**d**) O_2_ in air, (**e**) NH_3_ in air with pre-adsorbed O_2_, (**f**) H_2_O in air with pre-adsorbed O_2_. (**c**) Reference band diagram for the n-doped SnO_x_ nanopillars. The dotted lines represent the donor level (E_d_), the dashed line the intrinsic Fermi level (E_i_).

The interaction of target gas molecules with the oxide nanopillars follows the reaction channels widely discussed in literature for oxide materials (see SI, Eq. 1–4 and references S1–S10). The adsorption mechanism can in principle also depend on the environment where exposure to target molecules occurs. As the present sensors have been tested under different conditions (ultra-high vacuum, low vacuum - hereafter denoted as “vacuum” - and ambient air), it is worth discussing the observed behaviour at the light of the different working atmospheres. The main difference between the two environments (ambient air and vacuum) is the presence of oxygen. In air, O_2_ quickly adsorbs on the surface and provides a key feature for the interaction with target molecules. It is important to observe that upon exposure to ammonia in ambient air the sensing layer behaves as a p-type system (i.e. resistivity increases upon exposure to a reducing molecule, Fig. 3). In turn, in vacuum conditions a resistivity increase for oxidizing target molecules (O_2_) is observed, and a resistivity decrease is observed upon exposure to reducing molecules (CH_4_, H_2_, Fig. 1c). In this case the sensing layer behaves as an n-type semiconductor. The result obtained in vacuum conditions is expected, as both SnO_2_ and ITO are classified among n-type semiconductor oxide materials. The behaviour in air upon exposure to ammonia can be explained on the basis of the scheme proposed by Oprea *et al*.^34^. The authors suggest that an n-type semiconductor can display a p-type inversion layer upon O_2_ adsorption and therefore a weakly reducing gas can increase the inversion layer resistivity, as far as the inversion condition is maintained. Consistently with this finding, we observe that humidity has the opposite effect, i.e. humidity increase determines a resistivity drop. In this case, the concentration of water molecules is much higher than the NH_3_ case and the effect of reducing molecules could quench the inversion layer and restore the n-type behavior. The different testing conditions are depicted in Fig. 5.

Once band bending effects and the dual (n-type o p-type) operating condition has been elucidated, one is left with the effects of morphology and transport through the junctions.

Chemical sensing is about surface and interface interactions between the analyte molecules and the sensing material. Nanostructured sensors, for example made of nanotubes, nanowires or nanoclusters of SnO_2_, exhibit a high surface area-to-volume ratio, which strongly improves gas detection. The mechanism envisioned involves the adsorption, diffusion, electron transfer, as well as the change in the depletion zone related to the analyte molecule at the nanowire/surface junctions. In nanostructured materials, the sensitivity is usually related to the highly resistive nodes formed at the interfaces between different nanostructures. This was well demonstrated by Kolmakov *et al*.^35^ using photoemission microscopy on SnO_2_ nanowires between two metallic electrodes. They analyse the properties of individual SnO_2_ nanowires in a network of active elements in chemiresistor devices, showing the *in-situ* characterization of electron transport properties of mats of percolating nanostructures. It was shown that the transport quality depends on the interfaces between the nanowires. Therefore, reducing the number of interface junctions between the nanostructures as much as possible before reaching the electrodes is a likely way to increase the detection limit of the chemiresistor sensor. In our sample, the SnO_x_ nanostructures are vertical (see Fig. 1a), typically isolated, with the only interface being the one with the ITO Sn-reduced substrate. Since the nanopillars don’t touch each other enough to make a continuous path between the electrodes, the current flows in the Sn-depleted ITO film only. We thereby completely remove all resistive nodes between the nanopillars keeping only the interfaces with the substrate. We are therefore benefitting from the high surface area of the nanostructured SnO_2_, without the need of any deleterious resistive nodes in the resistive channel.

In the present case, high sensitivity is obtained by combining the surface reactivity with an efficient transport of charges from the nanopillars to the substrate. Molecules interact with the surface layer and charge flows through this layer to the substrate. The sensor quickly tracks the concentration variation and the response can be assumed to scale with concentration according to the calibration curve, providing high sensitivity. On the other hand, a fast recovery allows to quickly resumes the initial R_0_ conditions, avoiding bias in the response due to a long recovery time. The response time is estimated as the time occurring for the signal to rise from 10% to 90% of the overall signal change (ΔR). In our system the signal sampling is carried out with a frequency of 1/1.16 s^−1^. Therefore the response time measure is actually limited by the read-out frequency. Faster times may occur, but they are not detectable.

**Figure 6:**
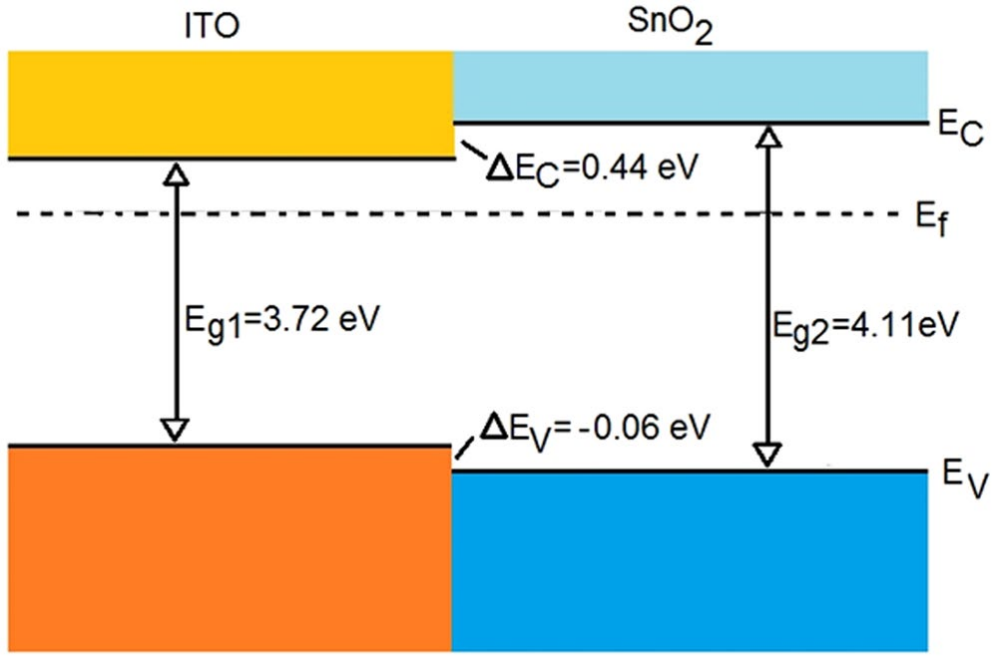
Energy band alignment at the ITO/SnO_2_ isotype heterojunction (adapted from ref.^37^).

The sensor resistance depends on possible potential barriers and on the number of conducting charges present in the large surface area of vertical nanopillars due to the analyte gas^36^. So, the mechanism that determines a greater response to the gases is twofold, involving both the morphology and the electronic properties (i.e. band alignment) of the heterojunctions established in the sensing layer.

(I) Morphology. The lack of contact points between the pillars excludes of percolation type mechanisms and therefore makes more efficient transport of the charges. This implies that the only interface involving the nanopillars is the interface between each single nanopillar and the ITO substrate. Sensing elements are well separated from each other allowing for chemisorption and fast desorption without trapping between pillars or inter-grain trapping as in polycrystalline thick and thin films. In these conditions, transport ultimately occurs across a single junction (SnO_2_-ITO) rather than being an inter-grain tunneling. This leads to efficient charge collection.

(II) Heterojunction. The interface above mentioned can be modelled as an heterojunction (HJ) between a SnO_x_ nanopillar and the ITO substrate (that contributes as an electrode to collect charge from the pillars). Assuming that close to the junction both systems are n-type materials, the heterojunction can be classified as an isotype junction and the band offset diagram for this type of system (ITO/SnO_2_) is discussed in ref.^37^. The energy band diagram, adapted from Tingliang *et al*.^37^, is shown in Fig. 6.

According to this diagram, a charge injection/extraction on the SnO_2_ side would end up in the ITO layer due to the favourable energy level mismatch both for holes and electrons. Indeed, the diagram presents a band alignment typical of a type I (i.e. straddling) HJ, where the material displaying a larger gap is SnO_2_ (4.11 eV), while the energy gap of ITO is 3.72 eV. With this type of junction, charges injected to the SnO_x_ sensing “layer” flow towards the substrate ITO because the minimum of the conduction band of SnO_x_ is above the minimum of the conduction band of the ITO. The same holds for holes injected in the SnO_x_ layer. They would flow to the ITO side as the ITO VB maximum is above the VB maximum of SnO_2_.

Room temperature operation is becoming an important topic in the field of gas sensing, as reported by a recent review^38^. According to the authors, 1D semiconductor nanostructures such as nanowires, nanofibers, and nanotubes can demonstrate appealing room-temperature sensing performances. Among metal oxides, In_2_O_3_-based nanostructures (i.e. nanowires and nanotubes) were found to operate at room temperature with a good sensitivity towards ammonia^39^, NO^40^, and H_2_S^41^, Likewise, also SnO_2_ nanostructures has shown remarkable RT sensitivity towards ethanol, CO, H_2_, and NO_x_^17,42^. In spite of these results, the mechanisms underlying RT operation still needs to be properly assessed.

The characteristic mechanism of metal oxide based sensors operating at high temperature is usually termed as combustive gas sensing effect, in which adsorbed analyte molecules become oxidised by interactions with pre-adsorbed surface oxygen ions^43^. In fact, those chemical surface interactions that lead to a combustive gas response are thermally activated. Thermal activation is not expected at low temperatures, and therefore evidence of significant low temperature sensing must have a different origin.

A discussion on alternative mechanism can be found in ref.^44^, where authors present sensors based on W_18_O_49_ nanowires with remarkable room-temperature sensitivity to NO_2_. The sensitivity is shown to decrease with temperature, rather than being higher, as usually found in metal oxide nanoparticles. The intensity of the sensor signals decreases drastically with temperature, up to being unresponsive to NO_2_ above 300 °C. The experimental results were found to be consistent with the model developed by Gurlo *et al*.^45^, thus indicating the formation of surface-trapped states NO_2_, including no evidence of dissociative NO_2_ adsorption. For the case of the present SnO_x_ nanorods, it is likely that the same mechanisms are at work, leading to a remarkable response at room temperature.

In addition to room temperature operation, several studies reported fast recovery in oxide nanofibers or, in general, in 1D nanostructures^46–48^.

In spite of the many studies, the mechanisms of fast recovery have not yet been assessed. However a common feature is represented by the unique, nearly 1D, structure of all these nanostructured oxides. Indeed, these systems usually display large specific surface areas, which may result in the absorption of large amounts of gas molecules. Charge accumulation layers are formed as oxygen molecules absorb to the surface of the materials. These accumulation layers may overlap with each other along the rod/fiber/pillar direction, producing continuous charge transfer channels. Charge mobility along this channels, together with a relatively low adsorption energy which favours molecules detachment, can be regarded as the mechanisms underlying the fast recovery. In other words, the fast recovery is determined by the capability to quickly drain the charge along the charge transfer channels before the weakly bound target molecules detach from the surface.

**Figure 7:**
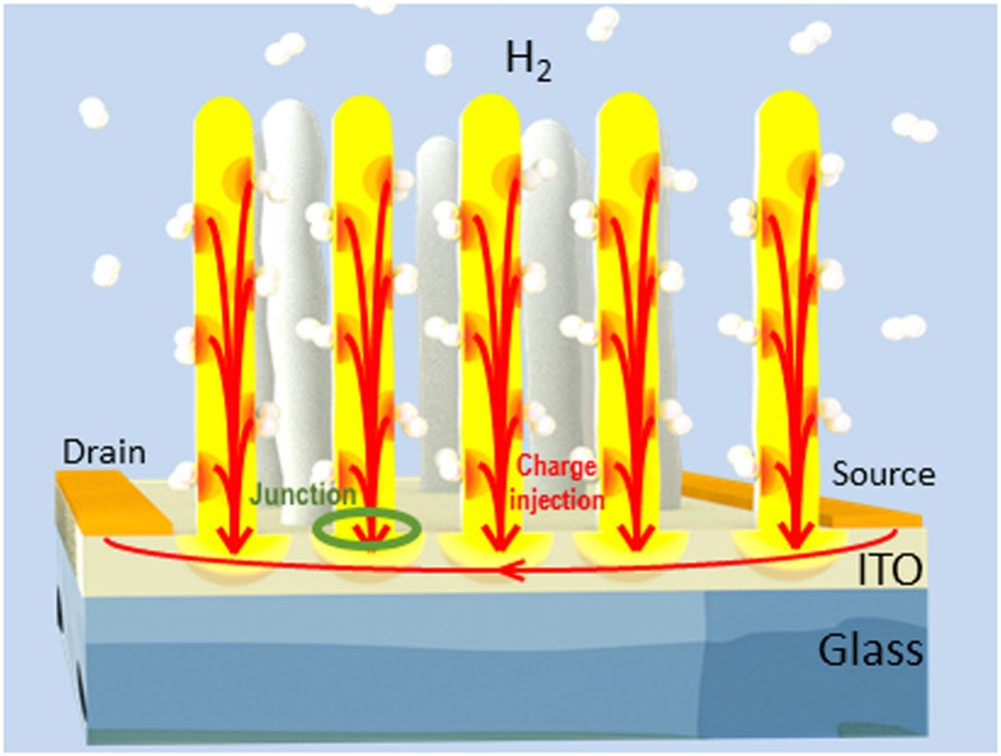
Schematic of the electron injection from the SnO_2_ nanopillars to the substrate upon the exposure to a reducing gas. The only junctions are at the nanopillar/ITO interface. Therefore, the current from source to drain does not have to pass through any junction.

Figure 7 schematically shows the overall charge flow in the sensor, from charge injection at the nanopillars surface down to the ITO sensing layer. A similar behavior was observed by Pan *et al*.^49^, which describe the effect as one-dimensional nanostructures gating the conducting channel in a two-dimensional ZnO nanocomb.

## Conclusions

We have reported a molecular architecture of SnO_x_ sensors that allows a detection of H_2_ below 5 ppm at RT and to a partial pressure of 10^−8^ mbar (10 ppt) at 230 °C. Using NH_3_ in air we observe a recovery time that is more than one order of magnitude faster than a commercial SnO_2_ sensor and faster than any other sensor detecting low concentrations (1 ppm) reported in literature. The combination of high sensitivity and fast recovery rates are explained by the unique nanostructuring of ITO in our device, which presents tin oxide vertical nanopillars on Sn-depleted ITO high-resistive layer.

The response to target molecules can be strongly determined by different environments (vacuum *vs* air). In vacuum the sensors behaves as n-type oxide semiconductor. Adsorption of O_2_ in air is likely to yield an inversion layer, which turns an n-type response into a p-type response of the SnO_x_ pillars. Charge transport occurs along the pillars surface until the SnO_x_-ITO interface is encountered. Here, charge transfer to the ITO layers occurs due to the favourable band alignment for both n-type and p-type carriers. We suggest that the remarkable sensitivity and the response rates of our system will enable portability for a wide range of applications.

## Methods

### Sample preparation

The sample is prepared starting from a ITO/glass substrate exposed sequentially to H_2_ and C_2_H_2_ at ~650 °C. This process forms vertical nanostructures of carbon filled with tin, as previously reported in detail^25^. The carbon shell can then be burned by heating the sample in oxygen at ~650 °C. Note that no further change to the structures are observed at this temperature, once the carbon is burned away. The final sensor is therefore made of a glass supports, coated with a Sn-depleted ITO film, ~120 nm thick, covered with vertical nanopillars of SnO_x_, see Fig. 1a,b. Ag metal electrodes are painted at both ends of the Sn-reduced ITO substrate.

### Gas sensing tests

The H_2_ measurements shown in Figs 1 and 2 are performed at the µ- & nano-carbon laboratory and SuperESCA beamline of the Elettra synchrotron in Trieste, where electrical measurements and X-ray photoemission spectra can be acquired in parallel. The base pressure in the chamber is ~10^10^ mbar and the dosed gas is measured with a hot filament pressure gauge. The chamber is pumped with turbo-molecular pumps.

The NH_3_ sensing measurements shown in Figs 3 and 4 are performed at I-Lamp laboratory in Brescia, the gas concentration is measured with a calibrated FIGARO TGS 2602 sensor. Both our and the Figaro sensors, are mounted on a specifically designed circuit exposing the sensors to a point-like source of NH_3_. In the same setup, humidity and temperature sensors are also mounted. Further details can be found in refs^50,51^.

## Electronic supplementary material

Supplementary Information
