# Supplementary material for: Improved recovery time and sensitivity to H2 and NH3 at room temperature with SnOx vertical nanopillars on ITO
Source: Sci Rep. 2018 Jul 3;8:10028. doi: 10.1038/s41598-018-28298-w (PMC6030158; doi:10.1038/s41598-018-28298-w)
Supplement: Supplementary file 1 — Supplementary Information [file 41598_2018_28298_MOESM1_ESM.docx]

Improved recovery time and sensitivity to H_2_ and NH_3_ at room temperature with SnO_x_ vertical nanopillars on ITO

*L. D’Arsié,^a,b,*^* *V. Alijani,^a^ S. T. Suran Brunelli,^a^ F. Rigoni,^c^ G. Di Santo,^a^ M. Caputo,^a^ M. Panighel,^a,d^ S. Freddi, ^c^ L. Sangaletti,^c^ and A. Goldoni^a,*^*

^a^Elettra – Sincrotrone Trieste S.C.p.A., s.s. 14 km 163.5 in Area Science Park, 34149 Trieste, Italy.

^b^Department of Engineering, University of Cambridge, Cambridge CB3 0FA, United Kingdom.

^c^Interdisciplinary Laboratory for Advanced Materials Physics and Dipartimento di Matematica e Fisica, Università Cattolica del Sacro Cuore, Brescia, Italy.

^d^Università degli Studi di Trieste, Piazzale Europa 1, 34127 Trieste, Italy.

^*^lorenzo.darsie@cantab.net and andrea.goldoni@elettra.eu

**SUPPORTING INFORMATION**

Figure SI.1 shows a zoom of the sample used in this study where it is possible to see in great details the pillars form. Figure SI.2 instead shows a substrate pretreated like the one in Figure SI.4 where, however, no nanostructures where grown. In both Figures SI.1 and SI.2, it is evident the nano-morphology and roughness structuring of the substrate. However by trying to use the substrate of Figure SI.2 as a sensor (the resistivity in vacuum was about 10 MΩ/sq) we observe only a variation of the order of 0.2-0.5% after the introduction of 1000 ppm of H_2_, CH_4_, O_2_, N_2_, CO_2_ per 100 s at RT.


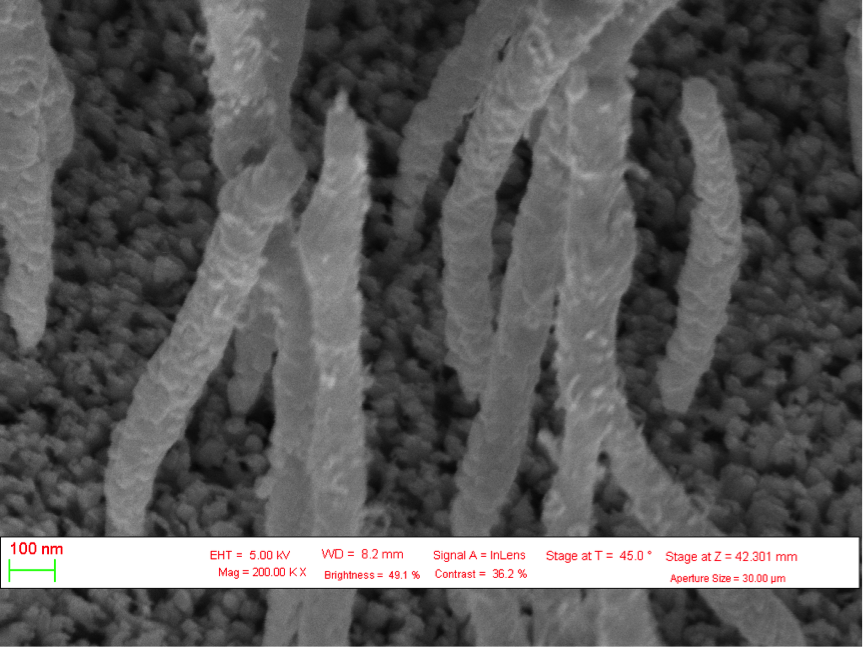


**Figure SI.1:** SEM image of SnO_2_ nanostructures on porous tin depleted ITO.


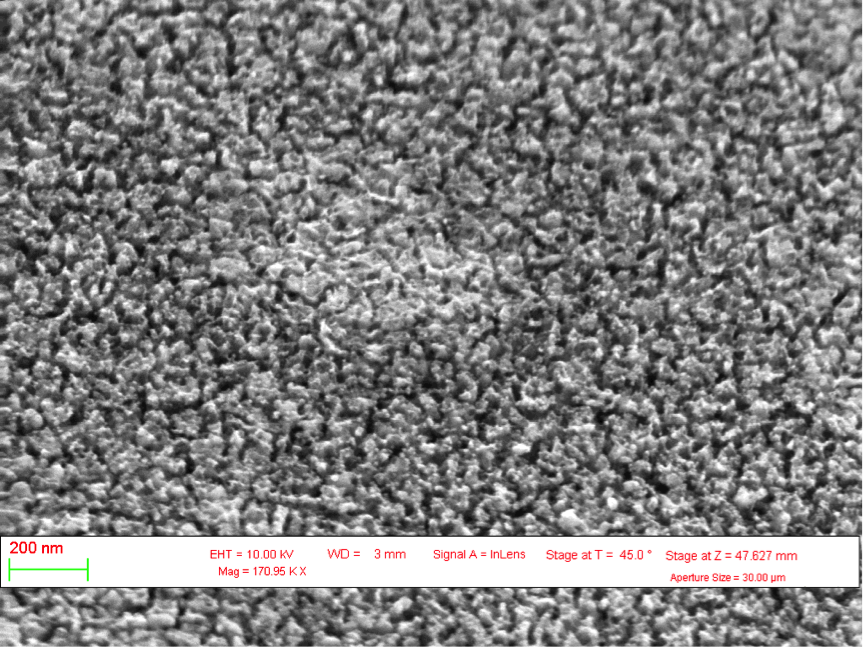


**Figure SI.2:** SEM image of porous ITO substrate after sample pretreatment, but without growth of nanostructures.

**Figure SI.3** demonstrates that the Sn 3d XPS spectra taken in real time during the exposure to 0.5 ppb of H_2_ of vertical SnO_x_ nanopillars sensor show a downward band bending of 0.40 eV (the hydrogen donate electrons and move up in the conduction band the Fermi level, so the core levels shift away from the Fermi level by the same energy)**.** The shift can be attributed to adsorption of small amounts of physisorbed H_2_. Subsequent exposure to vacuum causes an inversion of the band bending and the core levels revert to the initial state, indicating a release of the physisorbed H_2_. X-ray photoemission spectroscopy (XPS) measurements were taken at 650 eV with an energy resolution of 0.1 eV.


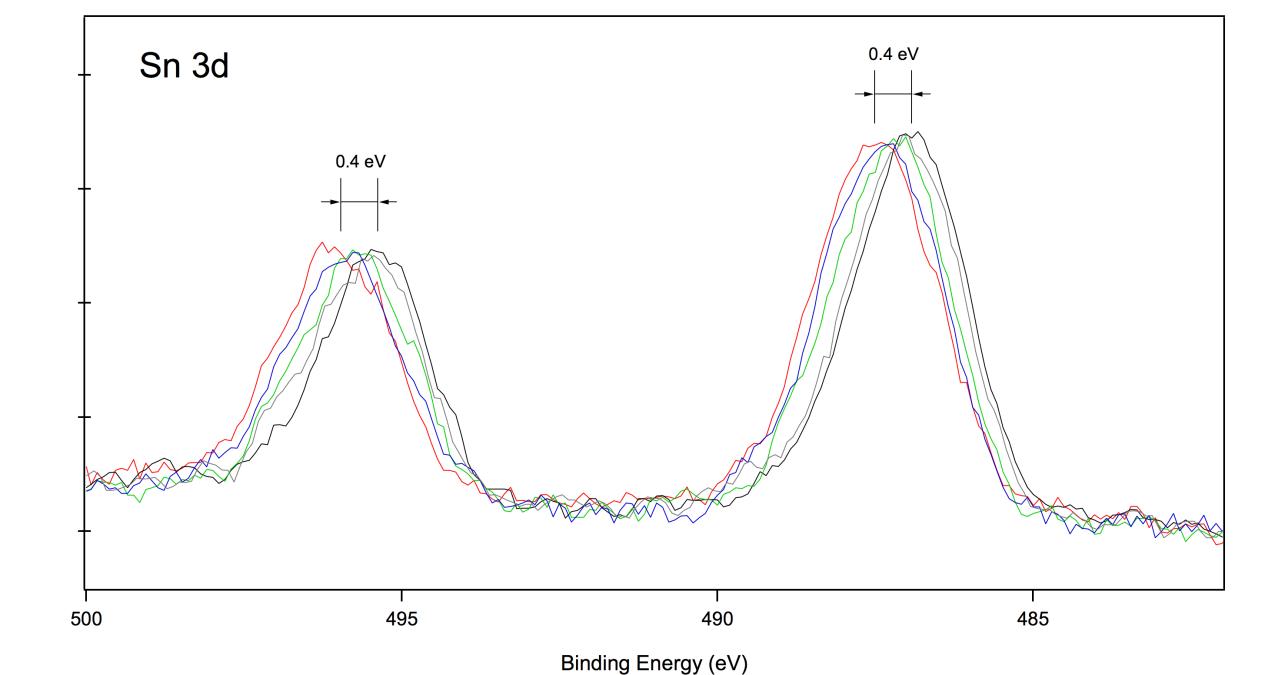


**Figure SI.3:** Sn 3d spectra taken in real time during the exposure to 0.5 ppb of H_2_. The spectra reported are the first (black), 15^th^ (gray), 25^th^ (green), 40^th^ (blue) and 50^th^ (red). The shift of the spectra while H_2_ is adsorbed is clearly visible.

Figure SI.4 displays the response of our sensor using four different samples. The plot shows a small variation between different samples that are within the experimental error. We can conclude that our measurements are reproducible.


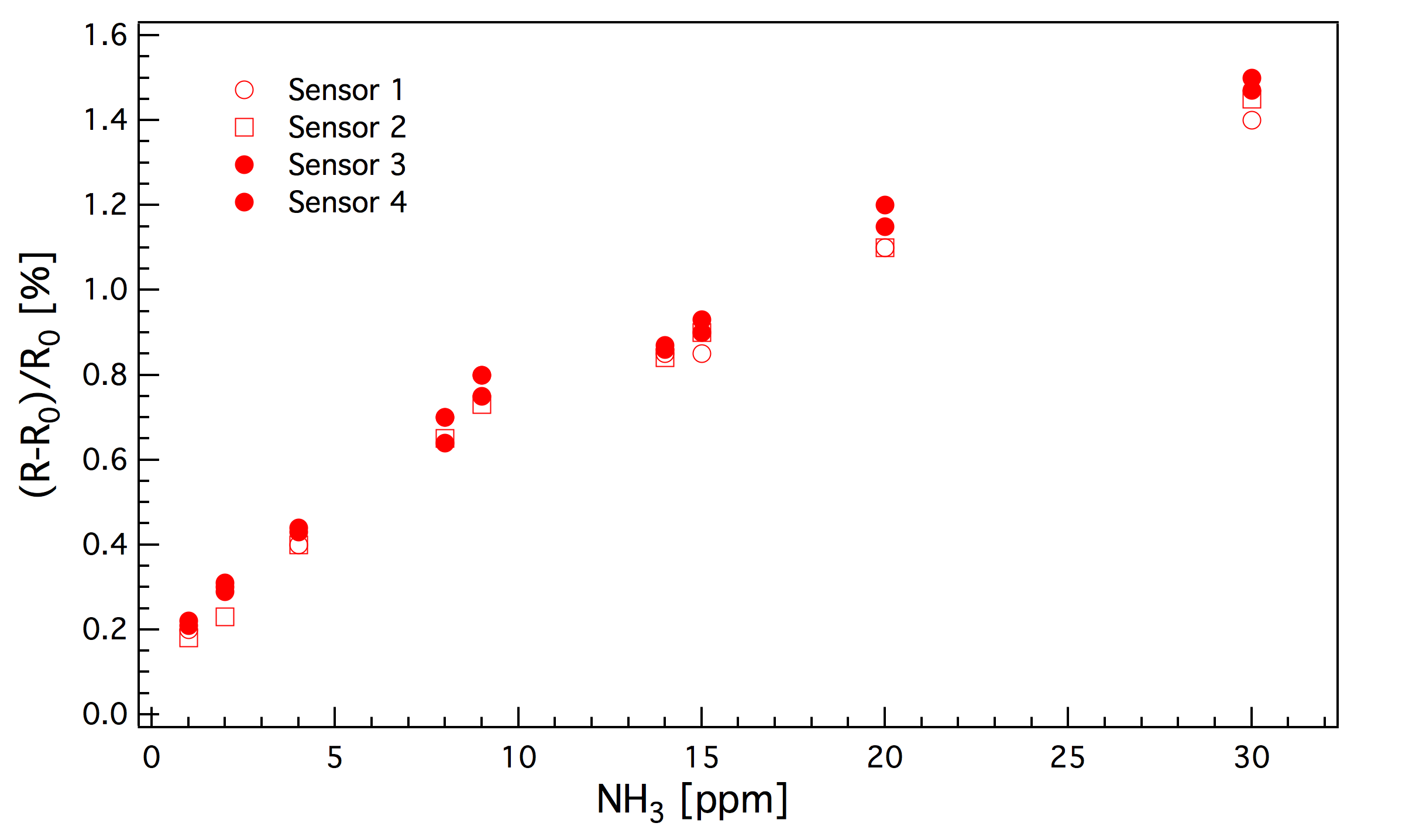


**Figure SI.4:** Response of four different substrates to the exposure of NH_3_.

Selectivity is attained by a two-fold point of view: on one hand the sensor is insensitive to some molecules used to test the device, such as acetone, alcohol etc. On the other hand the ΔR sign is a clear indication for discriminating different target molecules. Stability is provided by fast recovery, which avoids the contribution of bias background signals, due to quite slow recovery, that ultimately affect the baseline resistance (R_0_) and therefore the stability on a large time scale.

**Figure SI.5** shows that the sensor is not sensitive to acetone, 2-propanol, and sodium hypochlorite. Response to ethyl alcohol is quite low as compared to ammonia. All measurements have been carried out in ambient air.

**Figure SI.6** shows the response to ammonia tracked for long exposure times (250-300 seconds) and high concentrations (50-70 ppm). In spite of the prolonged interaction with large concentration of gas, full recovery is attained due to the fast recovery time.


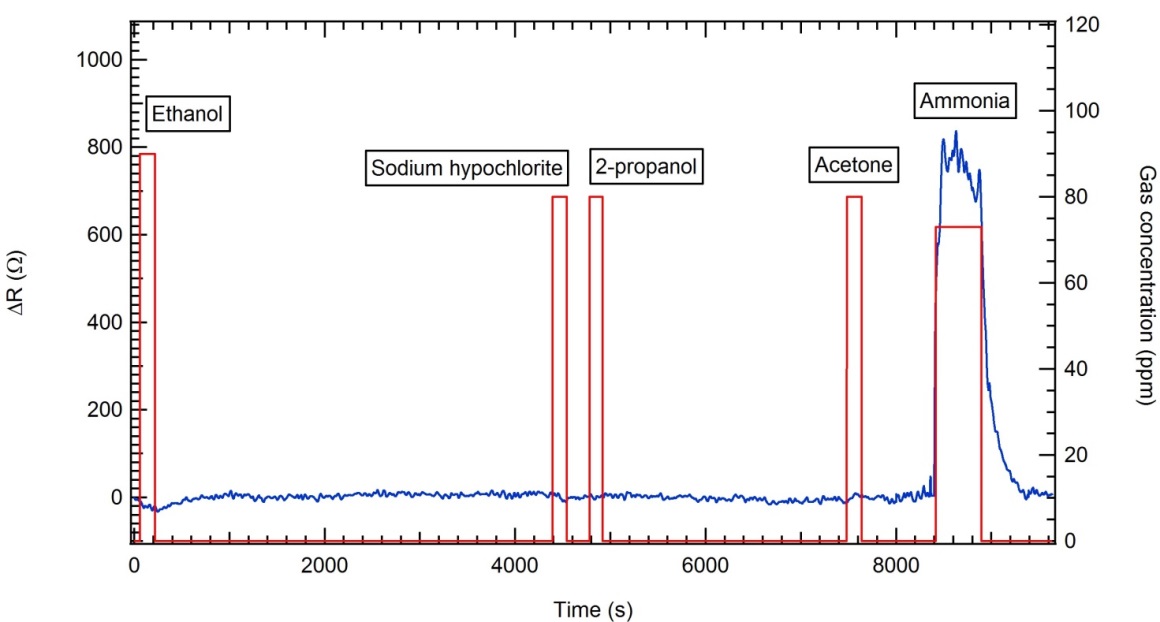


**Figure SI.5**. Response to different target gas molecules: ethanol, sodium hypochlorite, 2-propanol as compared to response to ammonia. All concentration in the 70-90 ppm range.


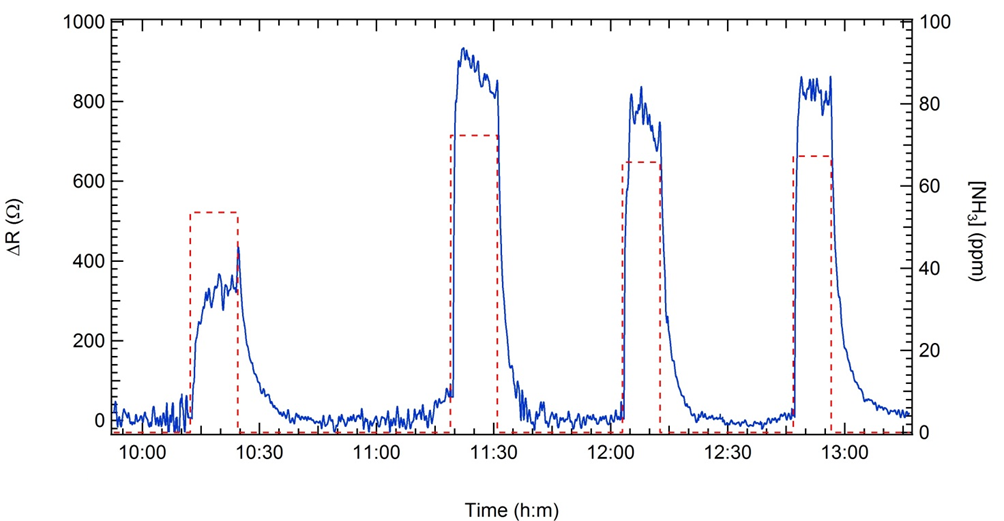


**Figure SI.6.** Response to ammonia multiple exposure; each exposure in the 250-300 s range; concentration in the 50-70 ppm range.

The response of the vertical SnO_x_ nanopillars sensors of NH_3_ strongly depends on the humidity. **Figure SI.7** shows that at a constant temperature of 23 °C the resistivity decreases when the humidity increases. This behavior is consistent with the n-type character of the nanosensor.  **Figure SI.8** (top graph) shows the sensor stability under common laboratory conditions; (bottom graph) temperature and relative humidity, monitored at the same time.


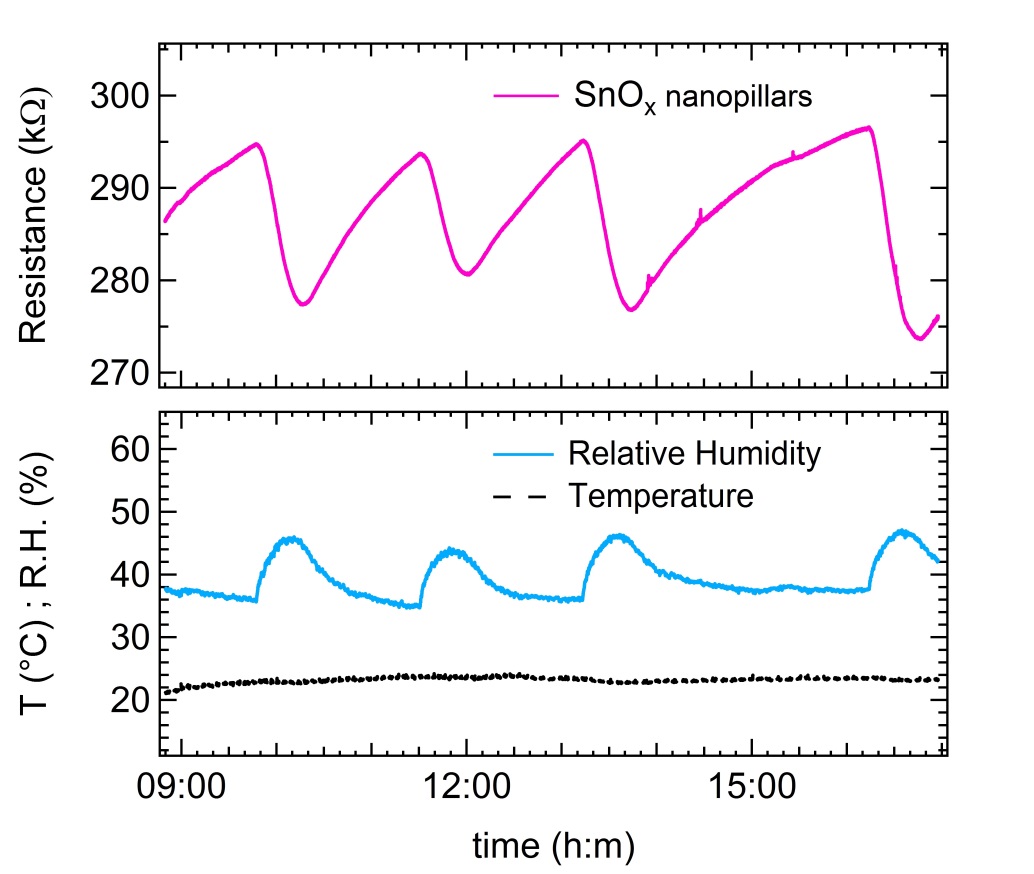


**Figure SI.7:** Air Resistance variation of the vertical SnO_x_ nanopillars sensors with relative humidity in at 23 °C during the day.


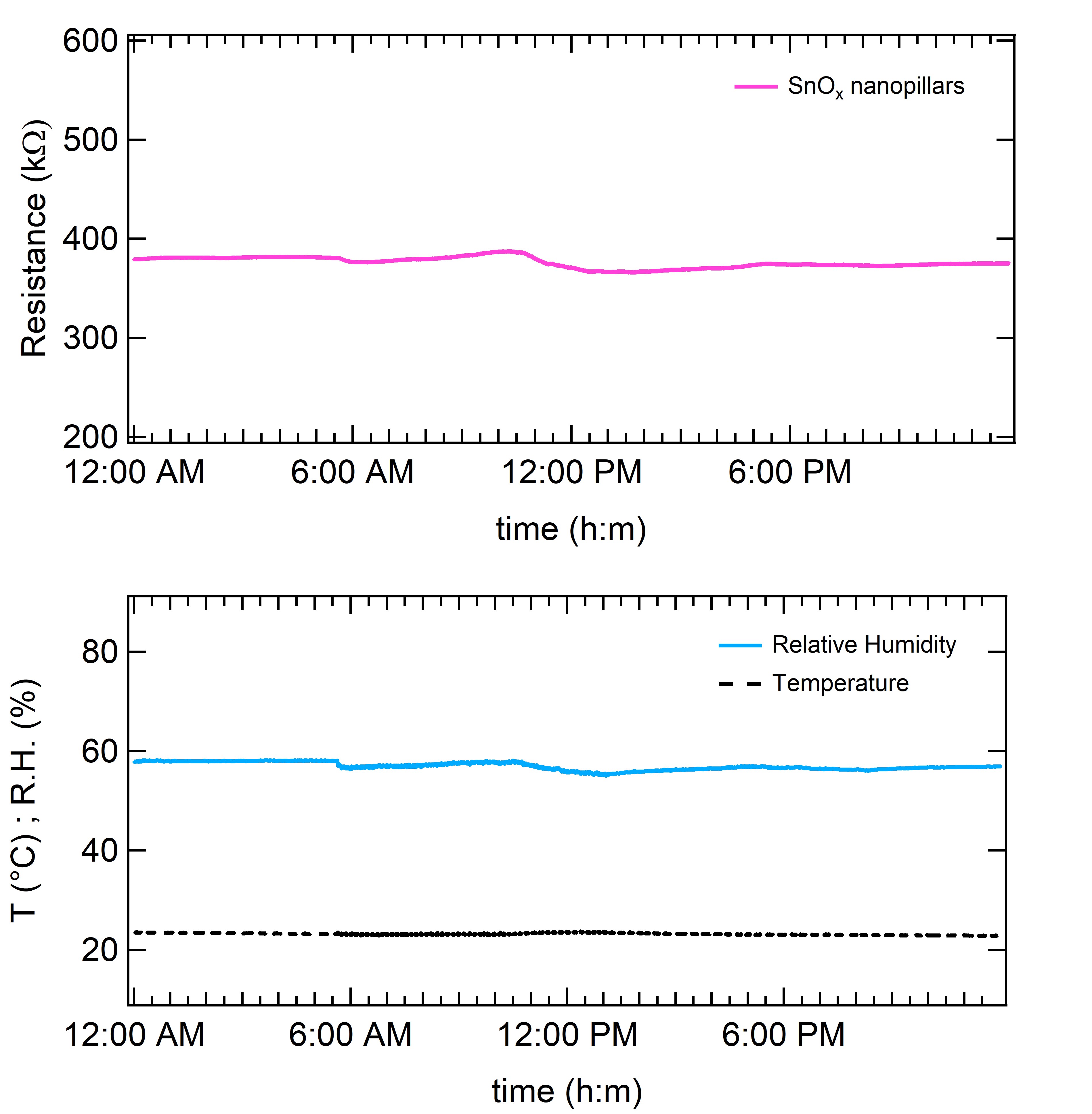


**Figure SI.8:** Sensor stability data, under the common laboratory environment (top graph). Temperature and relative humidity (graph below) are monitored in parallel at the same time.

**Figure SI.9** (top graph) shows the sensor response before and after 4 years of shelf-life and several sensing experiments.


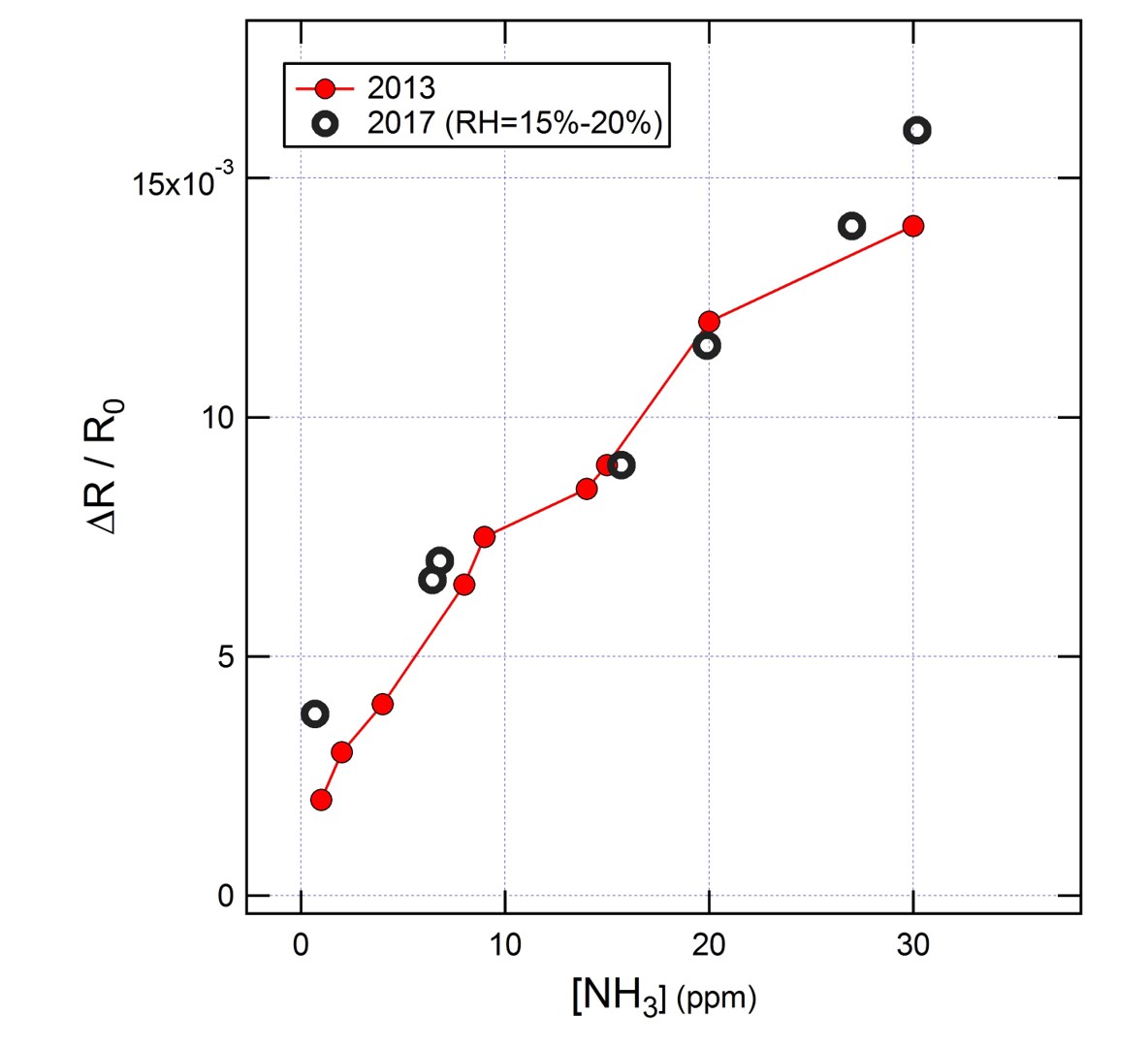


**Fig S.I.9** Response of the sensor at different NH3 concentrations. The figure shows the stability of the sensor after 4 years and several gas measurements. The behaviour is compatible, even though the base resistance (R0) that was 300 kΩ in 2013 and became 50kΩ in 2017.

In the following the possible surface interactions for selected gas molecules is discussed.

**Oxygen (O_2_)**

In the context of redox mechanisms, gases such as O_2_, NO_2_, CO_2_ which have the tendency to accept electrons from the metal oxide surface, are termed as oxidizing gases. Oxygen adsorbs quickly on metal oxide surfaces. The adsorption can be enhanced by increasing the operating temperature, by using dopants or by reducing the grain size [S1]. Below the temperature of 200 °C, O_2_ can accept one electron and above 200 °C, it can accept two electrons from the metal oxide surface, as summarized in Eqs. 1 and 2 [S2, S3].

O_2(gas)_+e^-^_(surface)_ → O_2_^-^_(adsorbed)_ [Eq.1]

O_2(gas)_+2e^-^_(surface)_ → 2O^-^ _(adsorbed)_, or O_2_^-^_(adsorbed)_+e^-^_(surface)_ → 2O^-^ _(adsorbed)_ [Eq.2]

**Hydrogen (H_2_)**

H_2_ molecules are adsorbed as protons on the thin film [S4]. Adsorption of protons on the surface, enhance the electron concentration, as shown in Eq. 3.

H_2_→ 2H^+^+ 2e^-^ [Eq.3]

**Ammonia (NH_3_)**

The lone pair of electrons of NH_3_ acts as an electron donor to the metal oxide, when reacted with the adsorbed oxygen ions on the surface by reverting the trapped electrons. The mechanisms that generate free electrons through the reaction of NH_3_ with oxygen ions was proposed by Nguyen et al. [S5] and among these mechanisms those described given in Eq. 4 are usually considered [S6-S10].

2NH_3_+3O^-^_(adsorbed)_ → N_2_+3H_2_O+3e^-^ , or 4NH_3_+3O_2_^-^_(adsorbed)_ → 2N_2_+6H_2_O+6e^-^ [Eq.4]

**References**

**S1.** Yamazoe, N. Sens. Actuat. B, 5, 7 (1991).

**S2.** Yamazoe, N., Sakai, G. & Shimanoe, K. Catal. Surv. Asia, 7, 63 (2003).

**S3.** Capone, S., & Siciliano, P. Encycl. Nanosci. Nanotechnol., 3, 769 (2004).

**S4.** Wurzinger, O. & Reinhardt, G. Sens. Actuat. B, 103, 104 (2004).

**S5** Nguyen, T., Park, S., Kim, J., Kim, T., Seong, G., Choo, J., Kim, Y., Beom, J., Kyu, T., Hun, G. & Shin, Y. Sens. Actuat. B, 160, 549 (2011).

**S6.** Singh, N., Shrivastava, S., Rath, S. & Annapoorni, S. Appl. Surf. Sci., 257, 1544 (2010).

**S7.** Rout, C., Hegde, M., Govindaraj, A. & Rao, C. Nanotechnology, 18, 205504 (2007).

**S8.**  Law, J. & Thong, J. Nanotechnology, 19, 205502 (2008).

**S9.** Wei, A., Wang, Z., Pan, L., Li, W., Xiong, L, Dong, X. & Huang, W. Chin. Phys. Lett., 28, 080702 (2011).

**S10.** Stankova, M., Vilanova, X., Llobet, E., Calderer, J., Bittencourt, C., Pireaux, J. & Correig, X. Sens. Actuat. B, 105, 271 (2005).
